# An Empirical Assessment of Transgene Flow from a *Bt* Transgenic Poplar Plantation

**DOI:** 10.1371/journal.pone.0170201

**Published:** 2017-01-13

**Authors:** Jianjun Hu, Jin Zhang, Xingling Chen, Jinhui Lv, Huixia Jia, Shutang Zhao, Mengzhu Lu

**Affiliations:** 1 State Key Laboratory of Tree Genetics and Breeding, Key Laboratory of Tree Breeding and Cultivation of State Forestry Administration, Research Institute of Forestry, Chinese Academy of Forestry, Beijing, China; 2 Collaborative Innovation Center of Sustainable Forestry in Southern China, Nanjing Forestry University, Nanjing, China; 3 Changchun Academy of Forestry, Changchun, China; Northeast Forestry University, CHINA

## Abstract

To assess the possible impact of transgenic poplar plantations on the ecosystem, we analyzed the frequency and distance of gene flow from a mature male transgenic *Populus nigra* plantation carrying the *Bacillus thuringiensis* toxin gene (*Bt* poplar) and the survival of *Bt* poplar seeds. The resultant *Bt* poplar seeds occurred at a frequency of ~0.15% at 0 m to ~0.02% at 500 m from the *Bt* poplar plantation. The germination of *Bt* poplar seeds diminished within three weeks in the field (germination rate from 68% to 0%) compared to 48% after three weeks of storage at 4°C. The survival rate of seedlings in the field was 0% without any treatment but increased to 1.7% under the addition of four treatments (cleaning and trimming, watering, weeding, and covering with plastic film to maintain moisture) after being seeded in the field for eight weeks. The results of this study indicate that gene flow originating from the *Bt* poplar plantation occurred at an extremely low level through pollen or seeds under natural conditions. This study provides first-hand field data on the extent of transgene flow in poplar plantations and offers guidance for the risk assessment of transgenic poplar plantations.

## Introduction

A major concern regarding the commercialization of transgenic forest trees is gene flow, through which transgenes may spread from transgenic trees to natural forests. Although gene flow is a very common natural phenomenon and an important process for evolution, the movement of transgenes from genetic modified plants to wild related populations may be considered undesirable and lead to undesirable environmental consequences, such as more aggressive weeds, germplasm loss, or increased non-target organism and biodiversity losses.

As long-lived plants, trees are exposed to the environment over a much longer time period than are annual crops [1, 2]. Poplar, as a fast-growing tree with 8–15 years in rotation, has been used for genetic transformation for both research and breeding [3, 4]. Cross-pollination of poplars in plantations with their wild relatives is of major concern due to the broad compatibility within and among species, even those belonging to different sections of *Populus* [5]. Several modeling and simulation methods have been proposed to measure the gene flow between poplar stands [6, 7], and co-dominant markers have been developed to monitor gene flow among species, even those belonging to different sections [8]. However, no empirical data on transgenic poplar plantations are available to address this issue.

Poplars play important roles in afforestation and timber supply worldwide. In China, approximately 7 million hm^2^ of poplar plantations have been established for shelterbelt and timber production [9]. However, insects create severe damage to the plantations and cause an estimated annual loss of millions of US dollars. The infected trees are usually sprayed with pesticide or cut down in order to control the infestation, resulting in serious economic and ecological consequences [10]. Genetic engineering provides a promising tool for breeding superior poplar clones with improved tolerance to insects, drought, and/or wood properties. For example, insect-tolerant transgenic *Populus nigra* with the *Bacillus thuringiensis* toxin (*Bt*) gene was planted for a field test in 1994 and proved effective in avoiding leaf-insect damage [11].

To date, transgenic poplars are not only released in field trials but also planted as a commercial crop in plantation forestry [12]. To develop poplars that were more tolerant to insect attacks, *Populus nigra* trees were transformed with *cry1Ac* in 1993 and have been field tested since 1994. *Bt* poplars were first commercialized in 2001 and occupied 490 ha in China up through 2014. Transgenic poplar plantations have effectively inhibited the fast spread of target insect pests and have significantly reduced the number of required insecticide applications [12].

In China, transgenic poplars are mainly planted in the northern areas where the climate is dry or semidry with an average precipitation from 100 to 600 mm concentrated in July and August. Poplar seed dispersal occurs from May to June and thus misses the rain season for favorable seed germination. Indeed, the successful establishment of a poplar plantation in these areas depends on the quality of seedlings planted and the availability of irrigation. In addition, no natural regenerated poplar stand has been found, and no natural regeneration of poplar plants has been observed around plantations. Taken together, it is apparent that transgenic poplar plantations will have little chance to spread transgenes into the natural forestry; this rationale prompted the National Forestry Administration in 2002 to grant permission for the commercialization of transgenic poplar clones in six provinces in China far from natural poplar stands. However, no empirical data have been collected to test and support this hypothesis.

In this study, we used the mature *Bt* poplar plantation at the Manas Plain Forest Station, Xinjiang Uygur Autonomous Region, to access possible gene flow from a transgenic poplar plantation through pollen and seeds. We would like to address the following questions: (1) How far does *Bt* poplar pollen travel and successfully fertilize other poplar trees in the local climate? (2) In a population of mixed non-transgenic and transgenic male poplars, are the seeds with the *Bt* gene produced according to the ratio of the two types of males? (3) What is the probability of *Bt* poplar seeds germinating and establishing under different conditions? The answers to these questions would provide the basis for understanding the safety issues surrounding the application of transgenic trees.

## Results

### Identification of the *Bt* transgene by PCR amplification of *Bt* fragments from transgenic leaves, pollen and hybrid seeds

**Fig 1:**
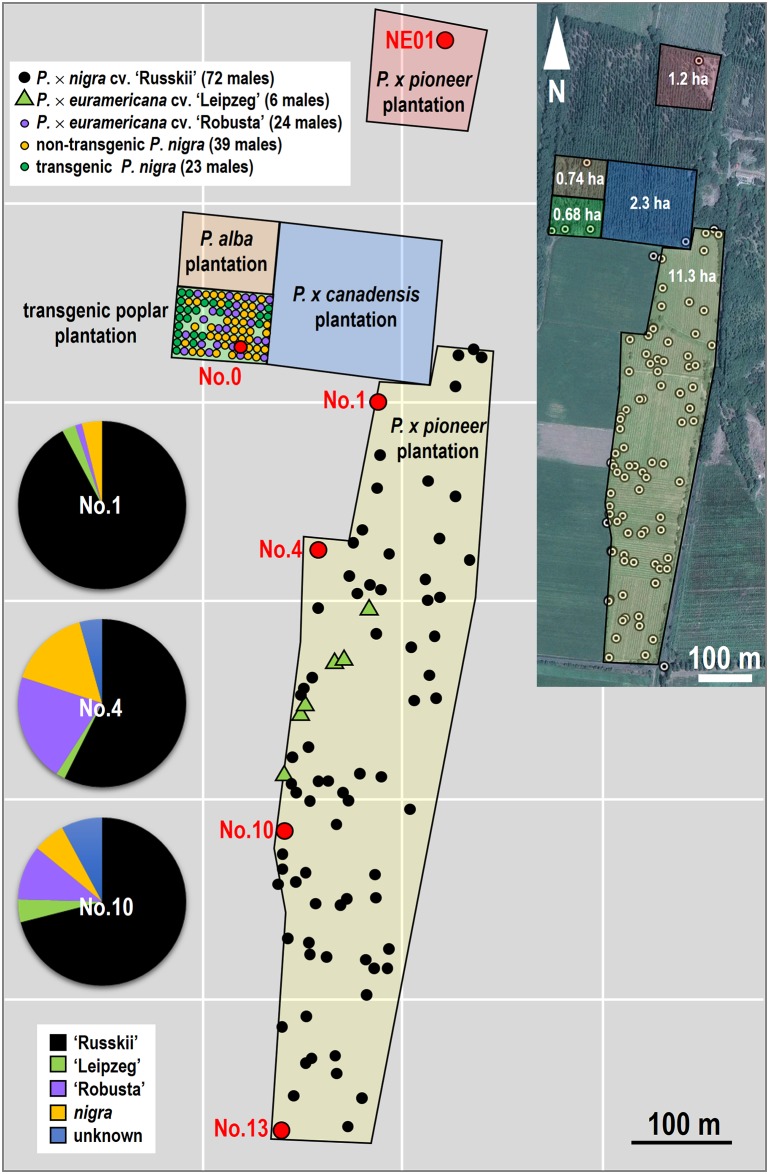
Sketched map of the sites of the transgenic poplar plantation (TPP) and the surrounding non-transgenic plantations. There were 23 *Bt* flowering transgenic male poplar trees and 63 non-transgenic male trees (39 non-transgenic *P*. *nigra* clonal trees and 24 *P*. × *euramericana* cv. ‘Robusta’ clonal trees) in the TPP, while there were 72 *P*. *nigra* cv. ‘Russkii’ (*P*. *nigra* cv. ‘Italica’× *P*. *nigra*) clonal trees and six *P*. × *euramericana* cv. ‘Leipzeg’ clonal trees distributed in the southeast ‘Pioneer’ plantation. The trees in the ‘Pioneer’ plantation that were sampled for seed collection were located in both the southeast (No. 0, No. 4, No. 10 and No. 13) and northeast (NE01). The distance from the sampling trees to the TPP are shown in Table 1. Pie graphs show the proportion of candidate paternity for the collected seeds at three sites.

**Fig 2:**
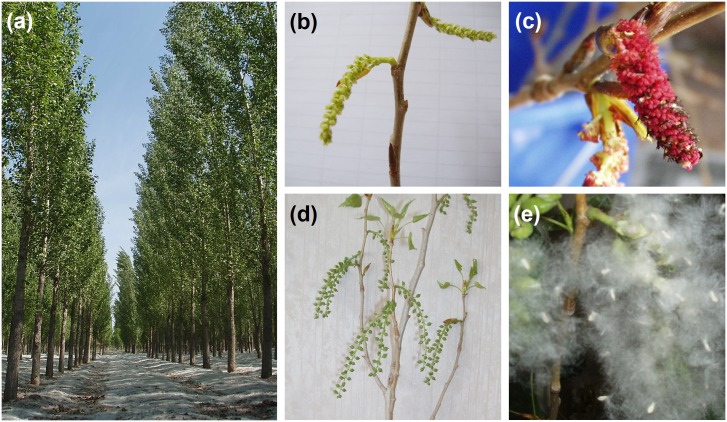
Female, male flowers, hybrid fruits and seeds of *P*. *nigra* cv. *‘*Pioneer’ and *Bt P*. *nigra*. **(a)** ‘Pioneer’ plantation. **(b)** Female flowers of the ‘Pioneer’ tree. **(c)** Male flowers of transgenic *P*. *nigra* (Cl. 222). **(d)** Fruits produced from a ‘Pioneer’ female branch crossed with pollen from *Bt* poplar. **(e)** Seeds of ‘Pioneer’ pollinated with *Bt* poplar.

**Fig 3:**
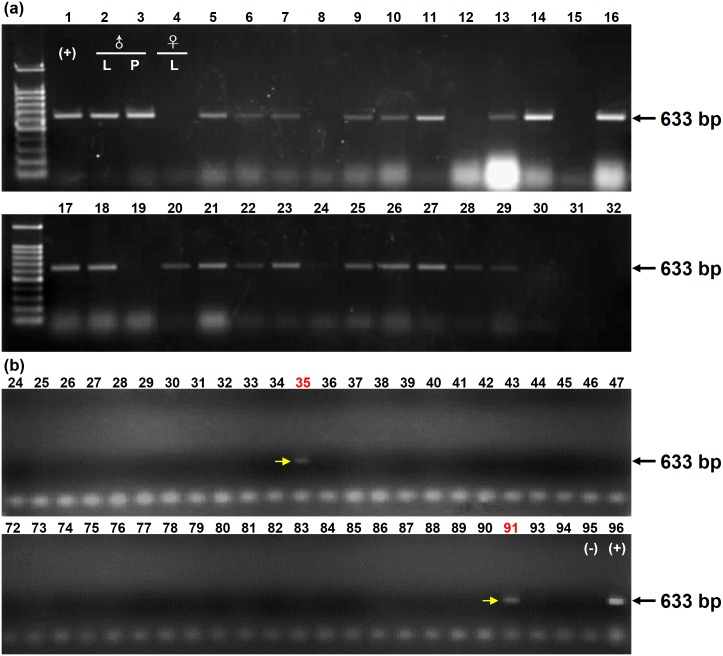
Agarose gel electrophoresis of products amplified from non-transgenic and transgenic poplars (a) and partial seeds collected at site No. 4 (b). **(a)** Lane 1, *Bt* plasmid as a positive control; Lane 2, leaves of *Bt* transgenic poplar; Lane 3, pollen of *Bt* poplar; Lane 4, leaves of non-transgenic poplar; Lanes 5–32, partial results of *Bt* detection from the offspring from controlled crosses between ‘Pioneer’ poplar and *Bt* transgenic poplar. **(b)** Lanes 24–47 and Lanes 72–94, partial results of *Bt* seed detection at site No. 4; Lane 95, leaves of non-transgenic poplar; Lane 96, *Bt* plasmid as a positive control. Lanes 35 and 91 are DNA samples showing positive *Bt* (arrow) detection. The amplified fragment was confirmed by sequencing (S2 Fig).

Seeds collected from non-transgenic CK3 (*P*. *nigra*) in the transgenic poplar plantation (TPP, Fig 1) and *P*. *nigra* cv. ‘Pioneer*’* trees (Figs 1 and 2A) at four sites (No. 4, No. 10, No. 13 and NE01; Fig 1) were used for DNA extraction and amplification of *Bt* fragments using gene-specific primers. The high cross compatibility between ‘Pioneer’ and *Bt* poplars was revealed by pollinating female flowers (Fig 2B) with pollen from transgenic male flowers (Fig 2C) to produce fruits (Fig 2D) and seeds (Fig 2E). The PCR products of the *Bt* fragment amplified from the leaves of female ‘Pioneer’ trees, the leaves and pollen of the transgenic male tree and the leaves of their progenies are shown in Fig 3A. The successful amplification of the *Bt* fragment from transgenic clones and their progenies indicated that the *Bt* gene still existed in the genome of transgenic poplars and could be transferred to its offspring by crossing. The amplified products of seeds collected from the ‘Pioneer’ trees at the four sites were also used to measure the probability of the transgene flow, and examples from site No. 4 are provided in Fig 3B.

### *Bt* seeds produced with pollen from the *Bt* poplar plantation

The collected seeds, ranging from 3,045 to 6,190 for each female tree at the four sites (No. 4, No. 10, No. 13 and NE01; Fig 1) in two adjacent years, were used to amplify the *Bt* fragment (Fig 3B) and measure the frequency of *Bt* seeds that occurred in the collected seeds; the results are listed in Table 1. *Bt* seeds occurred at rates of 0.16% and 0.15% (in the two years, respectively) at site No. 0 in the transgenic poplar plantation but only 0.05% and 0.07% at site No. 4 and 0.03% and 0.02% at site No. 10. Although more collected seeds were tested, no *Bt* gene was detected in the seeds at sites No. 13 and NE01. No. 13 was located 794 m southeast of the TPP, while NE01 was located 368 m northeast of the TPP. This result indicated that the number of detected *Bt*-containing seeds was related not only to the distance from the TPP but also the direction to the TPP.

**Table 1:** Summary of the proportion of seeds with *Bt* from sampled sites.

Sites	Distance to TPP	1^st^ Year			2^nd^ Year		
		Number of tested seedlings	DNA samples	*Bt* seedling (%)	Number of tested seedlings	DNA samples	*Bt* seedling (%)
**No. 0**	0 m	3045	609	0.16%	6190	1238	0.15%
**No. 4**	210 m	6180	1236	0.05%	4355	871	0.07%
**No. 10**	500 m	6055	1211	0.03%	4315	863	0.02%
**No. 13**	794 m	6060	1212	0%	4375	875	0%
**NE01**	368 m	6040	1208	0%	4735	947	0%

TPP, transgenic poplar plantation. After three days of germination, five seedlings were pooled as one sample for DNA extraction. The data were collected from two continuous years (2006–2007).

### Paternal assessment of the seeds collected from the sampled trees

To further analyze what potential pollen, other than *Bt* poplar pollen, contributed to the seeds collected from the plantation, 77, 115 and 116 seeds were collected from the sampled trees at sites No. 1, No. 4 and No. 10 for paternity analysis, respectively (Fig 1). Seeds from the female trees at No. 1, No. 4, and No. 10 were dominantly pollinated by *P*. *nigra* cv. ‘Russkii*’* male trees at rates of 92.21%, 57.39%, and 71.05%, respectively. This might be explained by the fact that there were 72 ‘Russkii’ trees distributed in the entire poplar plantation, accounting for 51% of the total male trees. In addition, 4.35% and 7.89% of the seeds from No. 4 and No. 10 were produced by unknown poplar males. These two sites were more open to the outside of the plantation, which may have been pollinated by male poplar trees other than those in the plantation, while the female tree at site No. 1 was slightly inside the plantation and thus not easily able to receive outside pollen.

### Germination ability and seedling survival of *Bt* poplar seeds under different conditions

The germination rates of seeds after storage at 4°C, at room temperature or under field conditions are summarized in Table 2. After three weeks of storage, the germination rates of *Bt* seeds stored at room temperature and in the field decreased to 7% and 0%, respectively, while a 48% germination rate was obtained for the seeds stored at 4°C for four weeks. It was determined that poplar seeds remained viable for only two weeks under natural conditions [13]. Therefore, there was no significant change in the viability of *Bt* poplar seeds. The higher germination rate of seeds stored at room temperature compared with that stored under field conditions indicates that besides temperature, other factors, such as exposure to sun light and air moisture, may also affect the germination ability of seeds.

**Table 2:** The average germination rate of *Bt* poplar seeds after storage.

Condition	The average germination rate after storage (%) (Mean±SD)			
	1 W	2 W	3 W	4 W
4°C	68±1.58 a	67±1.34 a	60±1.22 a	48±1.06
Room temperature	65±1.69 a	30±0.87 b	7±0.18 b	0
Field	12±0.32 b	3±0.21 c	0	0

W: week; SD: Standard deviation. Different letters represent significance at *P*<0.05 among the three conditions in each week. The average germination rate was 68±1.53 before treatment.

*Bt* poplar seeds were sown under field conditions with different treatments (see materials and methods). In the two plots without any treatments, no seedlings survived. For the six plots that were cleaned/trimmed, watered or covered with plastic film at the two sites, the rate of seedling establishment varied from 0.3 to 11.3% after one week but decreased to 0.2 to 3.5% after two months (Table 3). There was only a 0.3% germination rate of *Bt* poplar seeds at one plot due to water flow that formed by rain two days after the seeding.

**Table 3:** Seedling survival rate during eight weeks in the field after sowing.

Group	Condition ^a^	Survival rate ^b^			
		1 W	2 W	6 W	8 W
A	No action	0%	0%	0%	0%
B	C&T, Wa	3.0%	1.1%	0.2%	0.1%
C	C&T, Wa, We	6.6%	5.7%	2.5%	1.8%
D	C&T, Wa, We, C	8.8%	7.1%	2.5%	1.7%

^a^: Four conditions (Group A-D) were differentially combined with four treatments: cleaning and trimming (C&T), watering (Wa), weeding (We), and covering with plastic to maintain moisture (C).

^b^: For each condition, 2,000 seeds were used to evaluate the survival rate during eight weeks (W) after sowing in two independent areas.

## Discussion

The concern about transgene escape via the pollen or seeds of transgenic trees regards a possible negative effect on the natural forest ecological system, and this concern has hurdled the application of transgenic trees [14]. Thus, it is important to understand the pollen dispersal, seed production and seedling survival of transgenic trees. Previous studies have used established stands and simulation models to explore the consequences of introducing new genes into the environment. In this study, we take advantage of the commercialized insect-resistance transgenic poplar [15] and for the first time have provided empirical data to evaluate the possibility of transgene flow through pollen or seeds.

In natural populations, the possibility of introgression is especially high in poplars because reproductive barriers between species are weak. Meirmans et al. studied the rate of spontaneous hybridization from two poplar plantations into an adjacent natural population of *P*. *deltoides* and *P*. *balsamifera*. The significantly high rate of hybridization in the natural population suggests that small peripheral populations carry a higher risk of introgression [16]. Generally, biases in relative abundance can influence the direction of gene flow. Relative abundance may contribute to the asymmetric introgression of *P*. *nigra* genes, as this exotic species is much less abundant and is expected to contribute less to the pollen cloud than the native *P*. *balsamifera* in North America [17, 18]. These results indicate that the access of gene flow is very important and influenced by many factors that may lead to different genetic consequences in different populations. Therefore, the transgene flow of transgenic trees under field conditions needs to be studied.

Without commercialized transgenic tree plantations, it is not practical to assess transgene escape to conventional populations. The alternative would be to develop and exploit simulation models, such as STEVE and AMELIE, to gain insight into the gene flow in forest trees. Several models of pollen and seed dispersal have been proposed for forest trees [19–22]. For example, Kuparinen and Schurr investigated the rate of transgene escape in cases where the modified organism carried mitigation genes [21], and DiFazio used his model to predict transgene escape assuming long-distance dispersal as a common phenomenon [19]. Many results indicate that long-distance dispersal is extensive and that pollen dispersal curves are similar in populations with very different ecological and demographic characteristics [6]. Indeed, Roe et al. compared and contrasted exotic hybridization and introgression to the same processes occurring among native poplars in eastern Canada. In this native hybrid poplar zone, gene flow was asymmetric, with exotic alleles predominantly introgressing into *P*. *balsamifera* and hybrid formation among natives occurring primarily with the female *P*. *deltoides*. They also found that the majority of gene flow (more than 98%) was intraspecific [23, 24]. DiFazio et al. reported that the mean pollination distance for *P*. *trichocarpa* ranged from 140 to 1,100 m, with a strong dependence on the area sampled [20]. Pospísková & Sálková found that the effective pollination distance was 10 to 230 m within a *P*. *nigra* population along the Morava River [25]. The study of Rathmacher et al. showed that only a minor portion of gene flow occurred at distance beyond 1,000 m, and poplar seeds generally had shorter dispersal distances with a maximum distance of 500 m [26]. Bialozyt reported that most effective pollinations (75%) occurred within a distance of less than 1,000 m between native black poplar trees (*P*. *nigra*) and its commercial hybrid (*P*. × *canadensis*), and only a very limited proportion of effective pollinations occurred at distances greater than 2,000 m in central Germany [27]. Therefore, there was a wide range in the distance of pollen dispersal observed in poplar plantations based on these population studies and models. In our study, the results indicated that the pollen dispersal of *Bt* poplars occurred within 500 m in distance, which is close to the minimum limit of that predicted by the models or population analysis. The dispersal of *Bt* pollen was also affected by the wind direction during the flowering period in the spring. Due to the prevailing wind direction from the northwest to southeast in the study site, it is reasonable that no *Bt* pollen-pollinated seeds were detected in the northeast site (NE01), though the separation of an approximately 200 m-wide poplar plantation between the TPP and the site NE01 may have also limited *Bt* pollen dispersal.

The formation of a hybrid plant does not mean that a wild population will be established [28]. Hypocotyls develop within 6–8 h after moisture has reached the seed and the pappus has degraded [29], and the seedling cannot survive if conditions are not favorable for further development. Germination occurs exclusively on bare soil [30]. The results of this study revealed that transgenic poplar seeds lost germination ability under field conditions after three weeks but retained an almost unaffected germination ability at 4°C during long storage. Therefore, there is a crucial period of three weeks for the germination of transgenic seeds in the field. When sown at test sites without watering, no transgenic poplar seeds were germinated in the bare field, but a 3.5% seedling survival rate was obtained with watering, weeding and tillage. According to the study by Guilloy-Froget et al., successful germination of *P*. *nigra* seeds depends on a change to hydrated conditions [31]. Therefore, various factors, including soil, water, and weeds, will affect the germination and survival of seeds, and water seems to be the most important limiting factor. The significance of seed dispersal is highly dependent on plantation size [32]. In this study, the number of male transgenic poplar trees (23) was small, which might affect the fertilization of their pollen under competition with non-transgenic pollen from 141 males. As a result, only 0.16% and 0.15% *Bt* seeds from the control (No. CK3) were detected in the transgenic poplar plantation during the two years, respectively. The paternity analysis also supported this suggestion that the non-transgenic male trees dominated the pollen pool and would produce more non-*Bt* seeds; thus, transgenic seeds could not be readily produced under such conditions.

In summary, our study provides evidence that the pollen from transgenic trees travels a limited distance, and under the presence of a large number of non-transgenic males, the probability of *Bt* pollen successfully producing *Bt* progeny is quite rare. In addition, the transgenic seeds could not easily germinate and the seedlings could not easily survive in the dry areas of northern China. Therefore, it can be concluded that transgenes may not easily flow to the local poplar forestry based on this empirical data.

## Materials and Methods

### Plant materials and seed collection

The experimental site is located at Manas Plain Forest Station (N44°15^'^ 56^"^, E86°19^'^ 60^"^), Xinjiang Uygur Autonomous Region. The transgenic *P*. *nigra* plantation (TPP) was planted with two-year cuttings on approximately 0.68 ha of agricultural land (Fig 1) in 1994. The adjacent poplar plantations included a *P*. *alba* plantation in the north (0.74 ha), a *P*. × *canadensis* (a female clone) plantation in the east (2.3 ha) and a *P*. *nigra* cv. ‘Pioneer’ (*P*. *nigra* cv. ‘Italica’ × *P*. *nigra*, female) plantation in the southeast (11.3 ha) and northeast (1.2 ha). The field south of the transgenic poplar plantation was used for agriculture. There were 23 *Bt* flowering transgenic male poplar trees and 63 non-transgenic male trees (39 and 24 clonal trees of non-transgenic *P*. *nigra* and *P*. × *euramericana* cv. ‘Robusta’, respectively, in the TPP), while 72 and 6 clonal trees of *P*. *nigra* cv. ‘Russkii’ (*P*. *nigra* cv. ‘Italica’× *P*. *nigra*) and *P*. × *euramericana* cv. ‘Leipzeg’, respectively, were distributed in southeast ‘Pioneer’ plantation. No other male trees were planted at the experimental site. The details of each plantation are shown in S1 Table.

High crossability between transgenic *P*. *nigra* and *P*. *nigra* cv. ‘Pioneer*’* was observed in our pilot experiment (Fig 2), and the flowering time of these two clones was also compatible in the experimental site. The sampling trees in the ‘Pioneer’ plantation for seed collection were located in both the southeast (No. 1, No. 4, No. 10 and No. 13) and northeast (NE01). One female *P*. *nigra* tree control (CK3) in the TPP was also sampled. The plantation of *P*. × *canadensis* (female) was established in the same year (1994) as was the TPP. The sampling sites were chosen in the southeast plantation because the wind direction favored pollen dispersal in this direction in northern China. The period of pollination of the male trees was 5–6 days starting from April 8 at a temperature ranging from -5 to 23°C, with prevailing winds (0.2~10.7 m/s wind speed) from the northwest.

Seeds were collected from single trees at the above sites when dehiscing capsules began grouping in catkins in the spring of 2006 and 2007. Seeds were dried at room temperature, and the wool was removed by hand. Next, the seeds were put into envelopes in sealed plastic bags filled with silicon and stored in the cold. Germination rates were tested in triplicate with 100 seeds per replicate after cold storage for one, two, three, and four weeks. The seeds were sown on wet filter paper in Petri dishes, and the germination rates were determined on the fourth day after sowing. A seed was considered to have germinated with the appearance of two healthy cotyledons. To evaluate the germination of seeds and the survival of the seedlings in natural fields, we sowed 1,000 seeds in 16 plots with or without cleaning/trimming, weeding, watering or plastic film covering at two sites with 8 plots per treatment.

### Detection of *Bt* fragments by PCR

To detect *Bt* seeds, the collected seeds were sown on wet filter paper in a Petri dish and germinated at room temperature. After three days of germination, five seedlings were pooled as one sample for DNA extraction to reduce lab work (an extremely low *Bt* detection rate was observed in our pre-experiments) using the CTAB method. DNA concentration was estimated by comparing the brightness of the band with that of standard markers. Approximately 50 ng of DNA was used to amplify the *Bt* DNA fragment using gene-specific primers (5′-GAA TTC GCT AGG AAC CAA GCC ATT-3′ and 5′-AAG TAT ATC CAT CAA ATG TGG ACT-3′), and PCR was performed as previously described [33]. After 5 min at 94°C, the PCR reaction was carried out for 30 cycles at 94°C for 1 min, 55°C for 1 min, and 72°C for 1 min. After a final incubation at 72°C for 5 min, 5 μl of the PCR products was analyzed by electrophoresis in a 1.5% agarose gel and visualized under UV light after staining with EtBr solution. *Bt* seeds were used as control to check the fidelity of the PCR amplification.

### Paternity analysis

Four candidate paternal poplar clones (*P*. *nigra*, *P*. × *euramericana* cv. ‘Robusta’, *P*. *nigra* cv. ‘Russkii*’*, and *P*. × *euramericana* cv. ‘Leipzeg’) were identified in the plantations (Fig 1). Based on our previous study, the four paternal poplar could be effectively identified by four SSR loci (wpms04, wpms14, wpms18, and wpms20) (S1 Fig). To test the paternal source of seeds of the open-pollinated *P*. *nigra* cv. ‘Pioneer’, 308 seeds (from trees No. 1, No. 4, and No. 10) were analyzed using the four SSR loci. Primer sequences and PCR profiles were the same as described by van der Schoot et al. [34] and Smulders et al. [35]. Fragment analyses were performed on the QIAxcel System (QIAGEN, Germany). The Biocalculator software program was used to present the results as both simulated bands in gel images and peaks in electropherograms. The potential paternal parent of each seed was conferred by the appearance of the SSR patterns for each locus. The primers used in this study were shown in S2 Table.

## Supporting Information

S1 Fig The microsatellite patterns of the female parent and four possible male parents (‘Russkii’, ‘Leipzeg’, ‘Robusta’, or *nigra*, Fig 1) using four SSRs (wpms04, wpms14, wpms18, and wpms20). The red asterisks indicate the corresponding amplicon.
(TIF)

S2 Fig Alignment of amplified fragment and the full-length of *Bt Cry1Ac*. (TIF)

S1 Table Basic information for each plantation. (DOCX)

S2 Table Primers used in this study. (DOCX)
