# Supplementary figures and images for: An Empirical Assessment of Transgene Flow from a Bt Transgenic Poplar Plantation
Source: PLoS One. 2017 Jan 13;12(1):e0170201. doi: 10.1371/journal.pone.0170201 (PMC5234794; doi:10.1371/journal.pone.0170201)

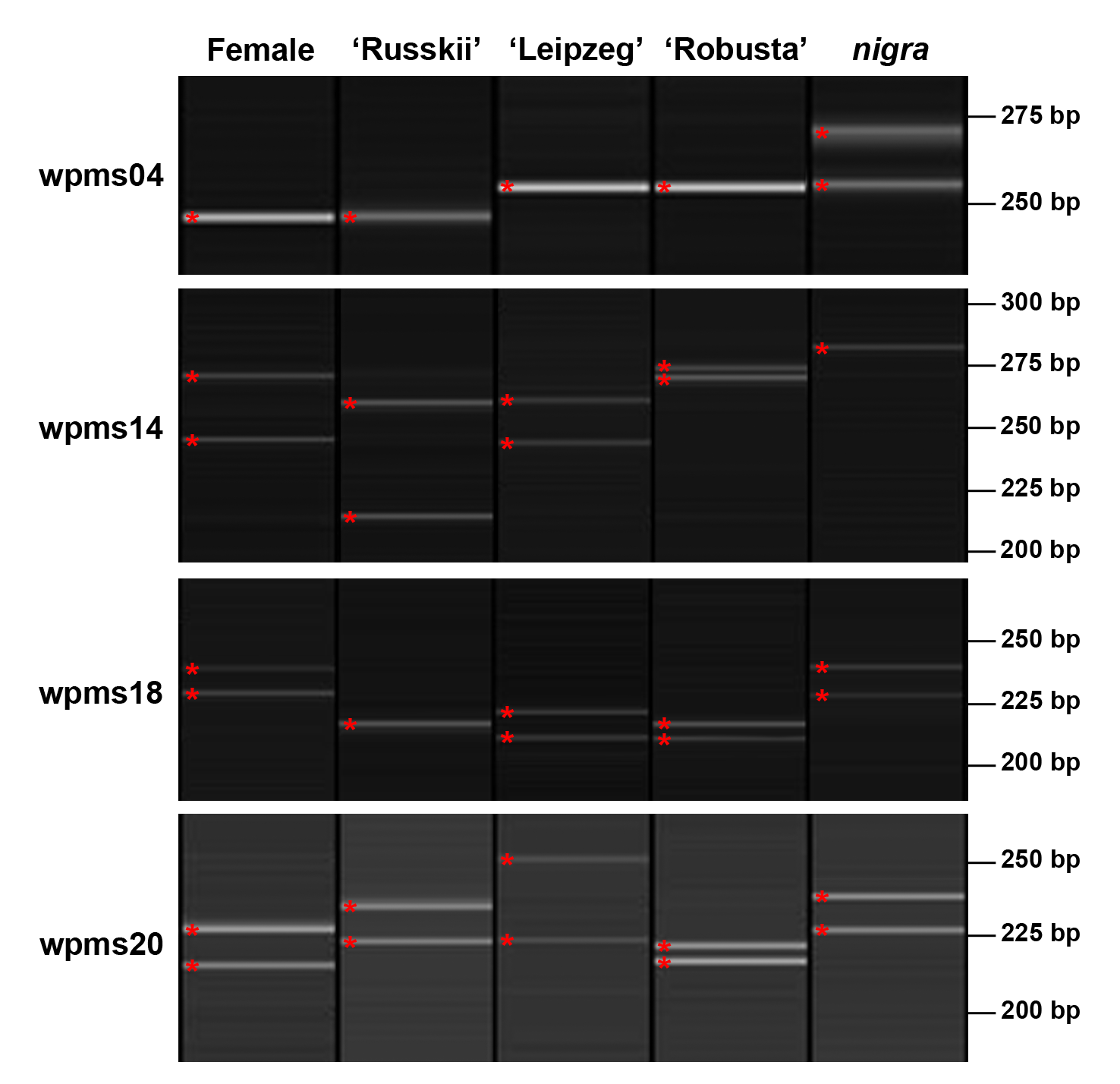

Supplement: S1 Fig — The red asterisks indicate the corresponding amplicon. (TIF) [file pone.0170201.s001.tif]

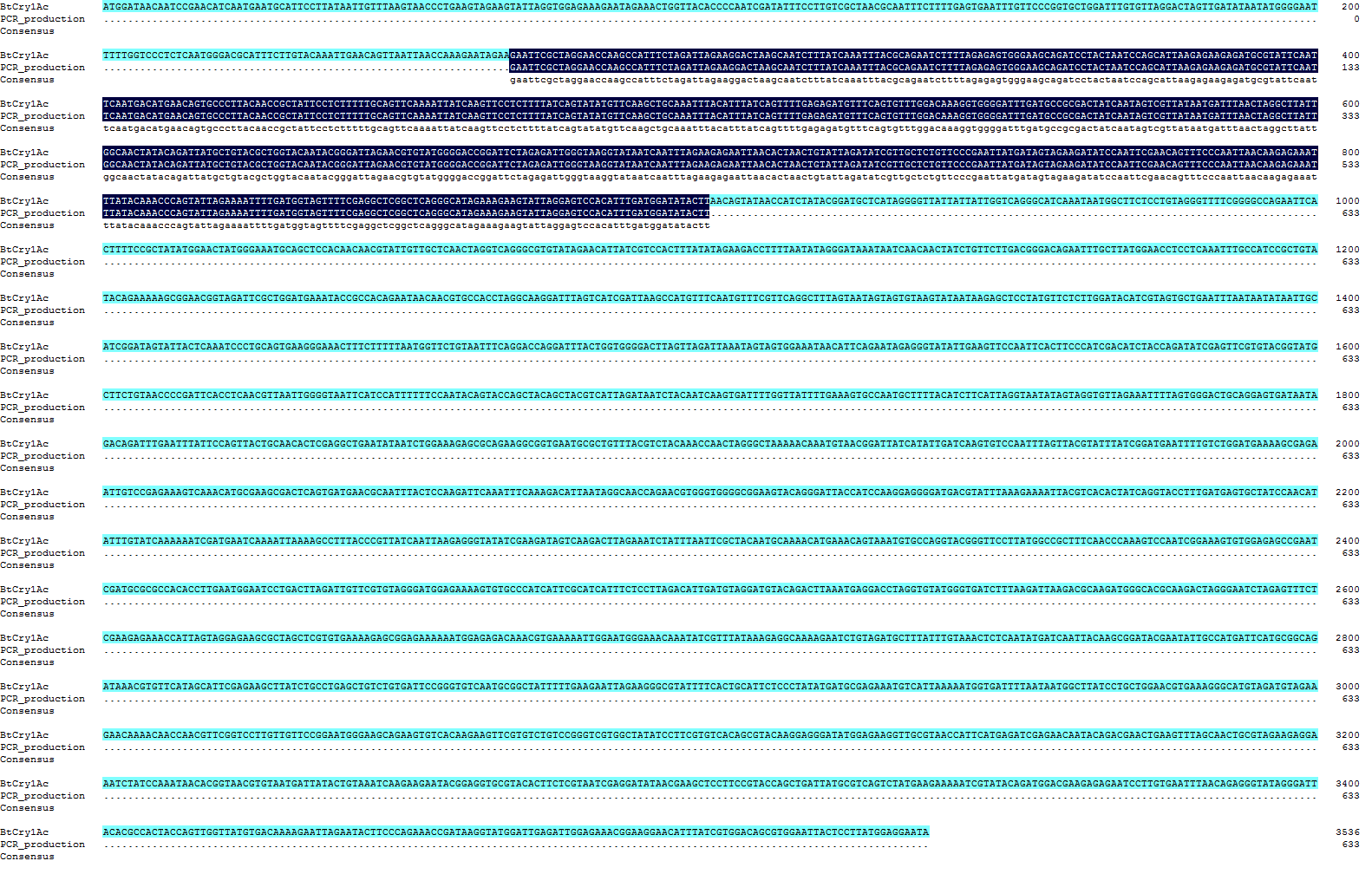

Supplement: S2 Fig — (TIF) [file pone.0170201.s002.tif]
