# Supplementary material for: An Empirical Assessment of Transgene Flow from a Bt Transgenic Poplar Plantation
Source: PLoS One. 2017 Jan 13;12(1):e0170201. doi: 10.1371/journal.pone.0170201 (PMC5234794; doi:10.1371/journal.pone.0170201)
Supplement: S1 Table — (DOCX) [file pone.0170201.s003.docx]

**Table S1** Basic information of each plantation.

| Plantation | Location | Area (ha) | Number of trees |
| --- | --- | --- | --- |
| Transgenic *P. nigra* plantation (TPP) | center | 0.68 | 264 |
| *P. alba* plantation | north | 0.74 | 292 |
| *P.* × *canadensis* plantation | east | 2.3 | 895 |
| *P*. *nigra* cv. ‘Pioneer’ plantation | southeast | 11.3 | 4392 |
| *P*. *nigra* cv. ‘Pioneer’ plantation | northeast | 1.2 | 468 |
