# Supplementary material for: An Empirical Assessment of Transgene Flow from a Bt Transgenic Poplar Plantation
Source: PLoS One. 2017 Jan 13;12(1):e0170201. doi: 10.1371/journal.pone.0170201 (PMC5234794; doi:10.1371/journal.pone.0170201)
Supplement: S2 Table — (DOCX) [file pone.0170201.s004.docx]

**S2 Table.** Primers used in this study.

| **SSR loci** | **Primer sequences (forward)** | **Primer sequences (reverse)** |
| --- | --- | --- |
| WPMS04 | TACACGGGTCTTTTATTCTCT | TGCCGACATCCTGCGTTCC |
| WPMS14 | CAGCCGCAGCCACTGAGAAATC | GCCTGCTGAGAAGACTGCCTTGAC |
| WPMS18 | CTTCACATAGGACATAGCAGCATC | CACCAGAGTCATCACCAGTTATTG |
| WPMS20 | GTGCGCACATCTATGACTATCG | ATCTTGTAATTCTCCGGGCATCT |
